# Linagliptin Protects against Endotoxin-Induced Acute Kidney Injury in Rats by Decreasing Inflammatory Cytokines and Reactive Oxygen Species

**DOI:** 10.3390/ijms222011190

**Published:** 2021-10-17

**Authors:** Tsung-Jui Wu, Yi-Jen Hsieh, Chia-Wen Lu, Chung-Jen Lee, Bang-Gee Hsu

**Affiliations:** 1 Division of Nephrology, Department of Medicine, Hualien Armed Forces General Hospital, Hualien 97144, Taiwan; ahreiwu@gmail.com; 2 Institute of Medical Sciences, Tzu Chi University, Hualien 97004, Taiwan; noirwen@gmail.com; 3 Division of Nephrology, Department of Internal medicine, Tri-Service General Hospital, National Defense Medical Center, Taipei 11490, Taiwan; 4 Division of Nephrology, Hualien Tzu Chi Hospital, Buddhist Tzu Chi Medical Foundation, Hualien 97002, Taiwan; hij@mail.tcu.edu.tw; 5 Department of Nursing, Tzu Chi University of Science and Technology, Hualien 97005, Taiwan

**Keywords:** dipeptidyl peptidase-4 inhibitors, linagliptin, acute kidney injury, endotoxic shock, reactive oxygen species, pro-inflammatory cytokines, AMPK pathway, NF-κB, conscious rats

## Abstract

Septic shock can increase pro-inflammatory cytokines, reactive oxygen species (ROS), and multiple organ dysfunction syndrome (MODs) and even lead to death. Dipeptidyl peptidase-4 (DPP-4) inhibitors have been proven to exert potential antioxidant and anti-inflammatory effects. We investigated the effects of linagliptin on endotoxic shock and acute kidney injury (AKI) in animal and cell models. In the cell model, linagliptin attenuated ROS by activating the AMP-activated protein kinase (AMPK) pathway, restoring nuclear-factor-erythroid-2-related factor (Nrf2) and heme oxygenase 1 (HO-1) protein, and decreasing pro-inflammatory cytokines (tumor necrosis factor alpha (TNF-α) and interleukin 1 beta (IL-1β)). In the animal model, 14-week-old conscious Wistar–Kyoto rats were randomly divided into three groups (*n* = 8 in each group). Endotoxin shock with MODs was induced by the intravenous injection of Klebsiella pneumoniae lipopolysaccharide (LPS, 20 mg/kg). Linagliptin improved animal survival without affecting hemodynamic profiles. In the histopathology and immunohistochemistry examinations of the rat kidneys, linagliptin (10 mg/kg) suppressed nuclear factor kappa-light-chain-enhancer of activated B cells (NF-κB) and inducible nitric oxide synthase (iNOS), decreased injury scores, and preserved E-cadherin expression from LPS damage. In conclusion, linagliptin ameliorated endotoxin-shock-induced AKI by reducing ROS via AMPK pathway activation and suppressing the release of TNF-α and IL-1β in conscious rats.

## 1. Introduction

Sepsis, an immune response to infection, has a complex pathophysiology and is associated with an increased risk of acute kidney injury (AKI). The annual global incidence of sepsis-associated AKI (S-AKI) is estimated to be approximately 1 per 1000 population [1]. Furthermore, 40%–50% of AKI patients hospitalized in the ICU are diagnosed with sepsis [2,3]. S-AKI is associated with increased odds of in-hospital mortality and longer hospital stay compared with AKI from other causes [2]. Renal recovery after S-AKI within 24 h after documentation of shock is reported to have a dramatically improved survival (hazard ratio 0.64; 95% CI 0.53–0.77) [4]. Although decreased global renal blood flow, ischemia-reperfusion damage, and following renal tubular epithelial cell (TEC) death have been recognized as the prevailing pathophysiology of S-AKI, accumulating evidence suggests that additional mechanisms must be at play [5,6]. Inflammatory mediators, with either pathogen- or damage-associated molecular patterns, are released and bind to toll-like receptors on immune cells or renal TEC surfaces during sepsis, which increases oxidative stress, the production of reactive oxygen species (ROS), and mitochondrial injury [7,8]. Consequently, the activation of dendritic cells, macrophage, and TECs is associated with increments of various pro-inflammatory cytokines, such as type 1 interferons (IFNs), CXC-chemokine ligand 2 (CXCL2), interleukin (IL)-1β, IL-6, IL-12, and tumor necrosis factors (TNFs) [9,10,11,12,13,14]. However, S-AKI therapy, especially preventive therapy, remains reactive and non-specific as the pathophysiology has yet to be fully elucidated.

Dipeptidyl peptidase-4 (DPP-4 or CD 26), which is an aminopeptidase, has been investigated as a membrane-spanning protein in numerous cell types and as a soluble protein in plasma. Currently, the anti-diabetic effect caused by the inhibition of DPP-4, which prolongs the action of glucagon-like peptide-1 (GLP-1), has been extensively investigated as the most important clinical application. Several potential mechanisms mediated by DPP-4 have been proven to improve endothelial function, decrease free radical production, and reduce inflammation in the lung ischemia-reperfusion injury mode [15,16]. DPP4i has also been demonstrated to increase the expression of vasoactive intestinal peptide (VIP) in ischemia-reperfusion injury in mouse orthotopic lung transplants [17] and inhibit xanthine oxidase activity to generate ROS in an LPS-sepsis model [18]. Linagliptin is a small molecule (molecular weight 473 daltons) and a highly potent competitive DPP-4 enzyme inhibitor that can work in various tissues [19,20,21]. Recently, linagliptin was demonstrated to attenuate organ (cardiac) dysfunction in diabetic patients with sepsis by inhibiting nuclear factor kappa-light-chain-enhancer of activated B cells (NF-κB) activation and associated excessive inflammation [22]. In this study, we aimed to investigate the effects of linagliptin on endotoxic-shock-induced AKI in the conscious rat model and identify the possible molecular mechanism.

## 2. Results

### 2.1. Linagliptin Restored the AMPK Pathway to Attenuate Endotoxin-Induced Overproduction of TNF-⍺, IL-1β, and ROS

**Figure 1:**
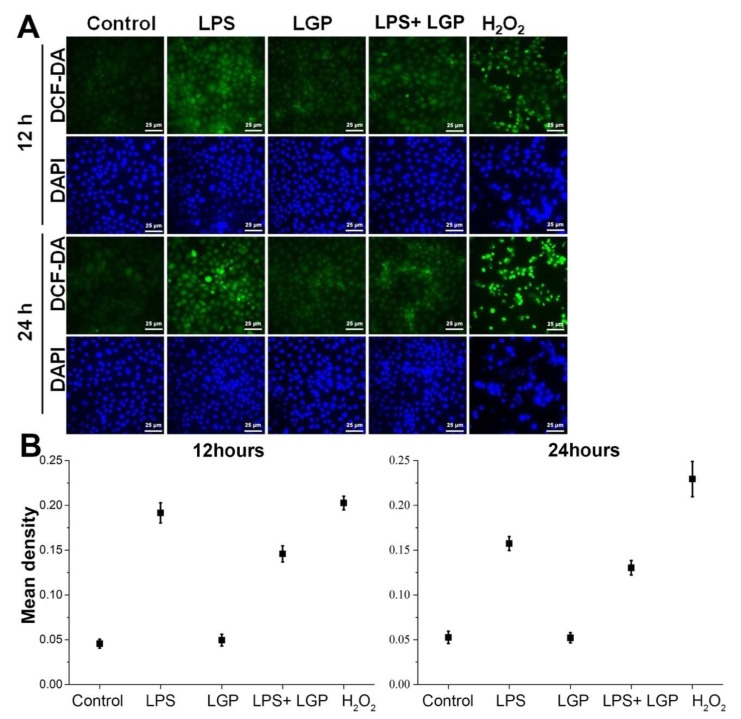
Reactive oxygen species (ROS) assay of NRK-52E cells after 12 or 24 h incubation with/without 10 μg/mL lipopolysaccharide (LPS) or 0.1 µg/mL linagliptin (LGP), using 2′,7′-dichlorodihydrofluorescein diacetate (DCF-DA) as a fluorescent probe and 4′,6-diamidino-2-phenylindole dihydrochloride (DAPI) to stain the nuclear acid. (**A**) 0.1 µg/mL linagliptin attenuated ROS induced by 10 μg/mL LPS in NRK-52E cells. (**B**) Semi-quantitative results of the ROS production by software ImageJ 1.47t.

We first tested whether linagliptin confers anti-oxidative capacity on rat kidney cell lines. In the ROS assay, linagliptin demonstrated better suppression of the intensity of DCF-DA fluorescence augmented by 12 h LPS treatment (Figure 1A,B).

**Figure 2:**
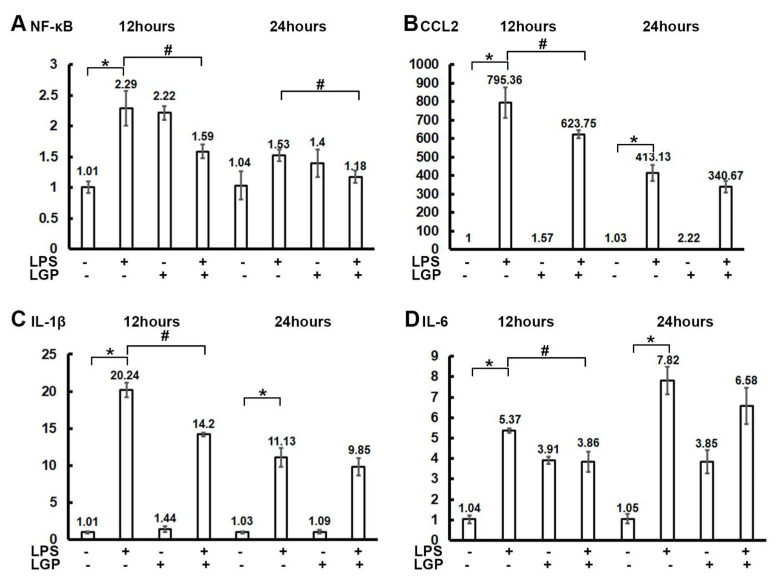
Quantitative real-time polymerase chain reaction (RT-qPCR) of NRK-52E cells after 12 or 24 h incubation with/without 10 μg/mL lipopolysaccharide (LPS) or 0.1 µg/mL linagliptin (LGP). The quantification results of RT-qPCR for (**A**) nuclear factor kappa-light-chain-enhancer of activated B cells (NF-κB), (**B**) chemokine ligand 2 (CCL2), (**C**) Interleukin -1β (IL-1β), and (**D**) IL-6. The relative normalized expression of target genes [2^−ΔΔCq^] were calculated using the reference gene β-actin as the internal control, sample “12 h control” for the rest of the groups in 12 h, and sample “24 h control” for the rest of the groups in 24 h. The error bar represents the standard error of the mean (SEM). * *p* < 0.05 for the LPS group compared with the Control group. # *p* < 0.05 for the LPS + Linagliptin group compared with the LPS group.

We next examined the mechanism of protection of linagliptin against ROS. In the quantitative real-time polymerase chain reaction (RT-qPCR) experiments on NRK-52E cells, the mRNA expressions of NF-κB, CCL2, IL-1β, and IL-6 were suppressed after treatment with linagliptin (Figure 2A–D).

**Figure 3:**
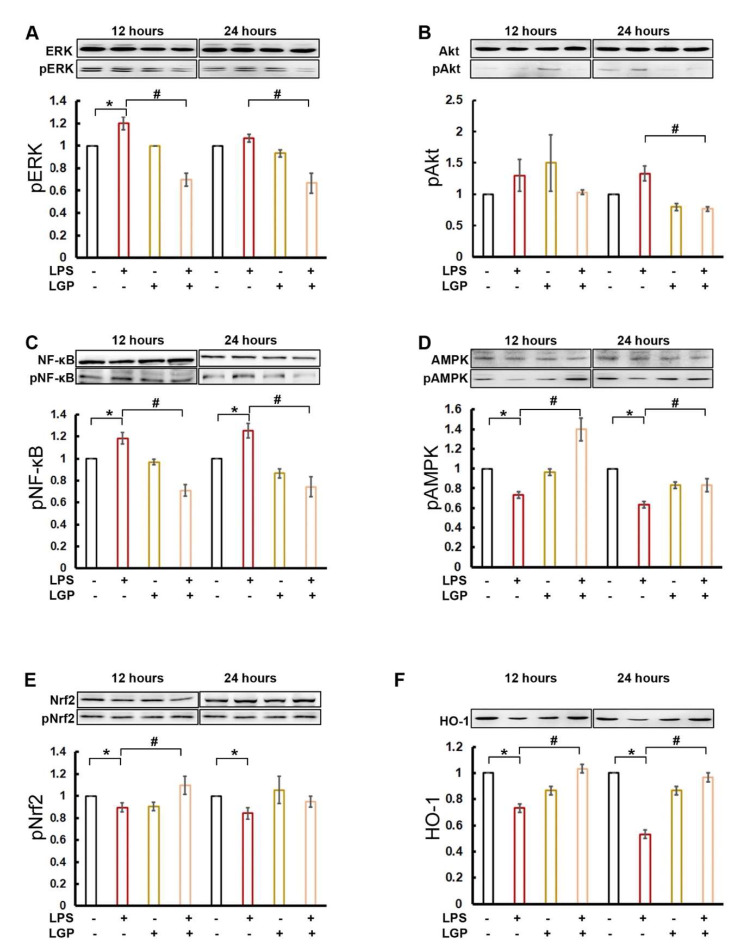
Western blotting of NRK-52E cells 12 or 24 h after incubation with/without 10 μg/mL LPS or 0.1 µg/mL linagliptin (LGP). Representative image and quantification results for inflammatory mediators include (**A**) extracellular-signal-regulated kinases (ERK) and phospho-ERK (p-ERK at tyrosine 204), (**B**) Protein kinase B (Akt) and phospho-Akt (serine 473), (**C**) NF-κB p65 and phospho-NF-κB p65 (at serine 536), (**D**) AMP-activated protein kinase (AMPK) ⍺1/2 and phospho-AMPK ⍺1/2 (at threonine 183 and threonine 172, respectively), (**E**) Nrf2, phosphor-Nrf2 (at serine 42), and (**F**) HO-1. The band of each sample was quantified by software ImageJ 1.47t, and all results were normalized to the value of the control β-actin. * *p* < 0.05 for the LPS group compared with the Vehicle group. # *p* < 0.05 for the LPS + Linagliptin group compared with the LPS group.

In the Western blot, the protein levels of the phosphorylated form of extracellular-signal-regulated kinases (ERK), Akt, and NF-κB were decreased after 12 h and 24 h co-treatment with LPS and linagliptin (Figure 3A–C), whereas the levels of phosphorylated AMPK, Nrf2, and HO-1 were significantly restored with 12 h and 24 h co-treatment with linagliptin and LPS (Figure 3D–F).

### 2.2. Linagliptin Attenuated Endotoxic-Shock-Induced multiorgan Damages, Inflammatory Biomarkers, and Survival in a Rat Model 

**Figure 4:**
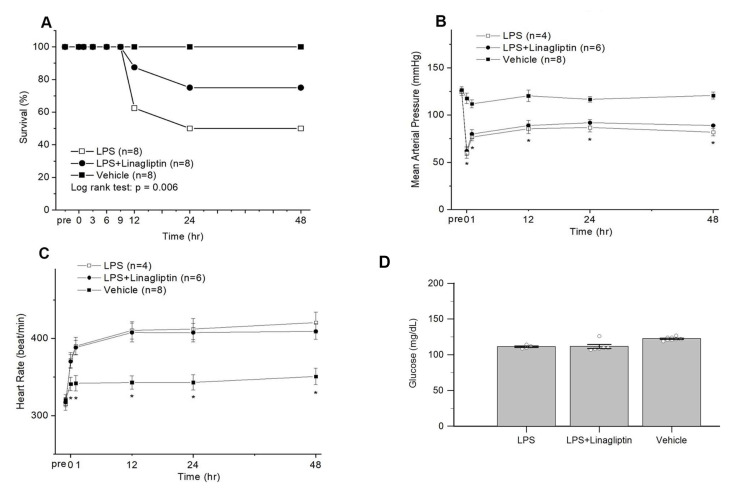
Changes in (**A**) survival curves, (**B**) mean arterial pressure, (**C**) heart rate, and (**D**) glucose after endotoxic shock in conscious rats. * *p* < 0.05 for the LPS group compared with the Vehicle group. Vehicle (*n* = 8), LPS (*n* = 4), and LPS + Linagliptin (*n* = 6) groups at 48 h after LPS administration.

We further examined whether the protective effects of linagliptin could be translated into animal survival from endotoxin-induced septic shock. All rats were alive 9 h after the induction of endotoxic shock. However, 2 and 4 rats died 12 h after the induction of endotoxic shock in the LPS and LPS + Linagliptin groups respectively. The survival rate at 48 h after the induction of endotoxic shock was 50% for the LPS group, 100% for the Vehicle group, and 75% for the LPS + Linagliptin group (Figure 4A). The mortality rate in the LPS + Linagliptin group was significantly lower than that in the LPS group (log-rank test; *p* = 0.006). In the survivors in LPS and LPS + Linagliptin groups, hypotension with tachycardia occurred within 30 min after LPS administration and blood pressure remained consistently lower than that in the Vehicle group (Figure 4B,C). No significant difference was observed in the serum glucose levels of the survivors in each group at 48 h after the induction of endotoxic shock (Figure 4D). 

**Figure 5:**
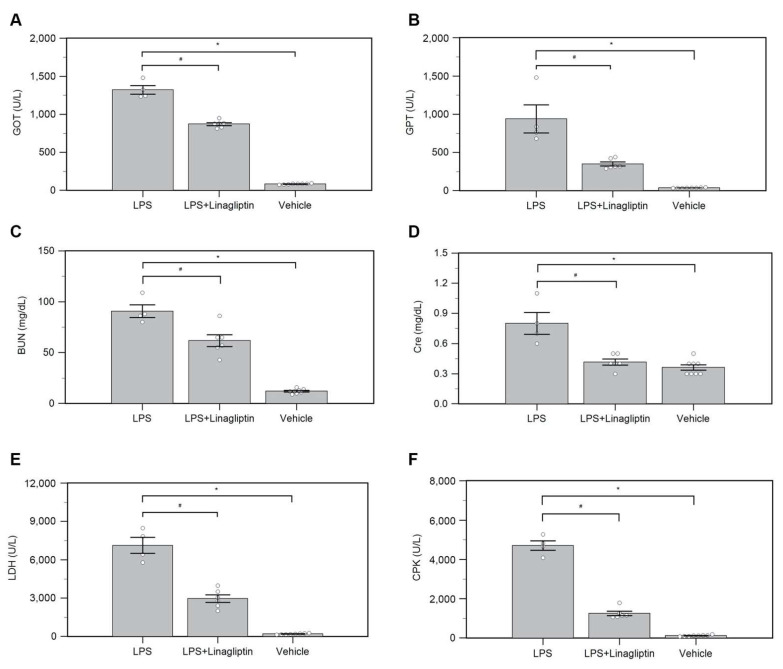
Changes in serum (**A**) glutamic oxaloacetic transaminase (GOT), (**B**) glutamic pyruvic transaminase (GPT), (**C**) blood urea nitrogen (BUN), (**D**) creatinine (Cre), (**E**) lactic dehydrogenase (LDH), and (**F**) creatine phosphokinase (CPK) after endotoxic shock in conscious rats. * *p* < 0.05 for the LPS group compared with the Vehicle group. # *p* < 0.05 for the LPS + Linagliptin group compared with the LPS group. Vehicle (*n* = 8), LPS (*n* = 4), and LPS + Linagliptin (*n* = 6) groups at 48 h after LPS administration.

**Figure 6:**
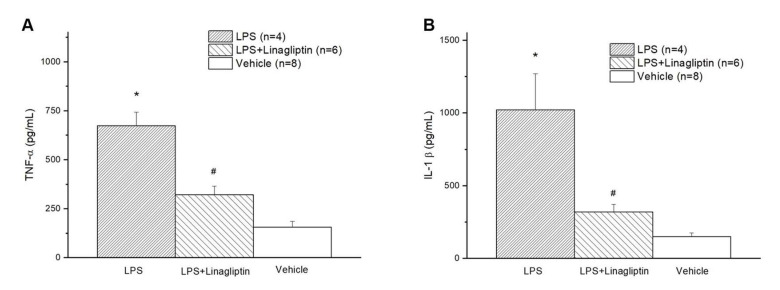
Changes in serum (**A**) tumor necrosis factor-α (TNF-α) and (**B**) interleukin-1β (IL-1β) after endotoxic shock in conscious rats. * *p* < 0.05 for the LPS group compared with the Vehicle group. # *p* < 0.05 for the LPS + Linagliptin group compared with the LPS group. Vehicle (*n* = 8), LPS (*n* = 4), and LPS + Linagliptin (*n* = 6) groups at 48 h after LPS administration.

In the Vehicle group, endotoxic shock induced multiorgan damage with elevated serum GOT, GPT, BUN, Cre, LDH, and CPK levels (Figure 5A–F) in the blood biochemical tests, as well as serum inflammatory biomarkers, TNF-α and IL-1β (Figure 6A,B). Treatment with linagliptin attenuated multiorgan damage and production of inflammatory cytokine caused by LPS (Figure 5A–F and Figure 6A,B).

### 2.3. Linagliptin Attenuated Endotoxic-Shock-Induced Renal Tubular Damages 

**Figure 7:**
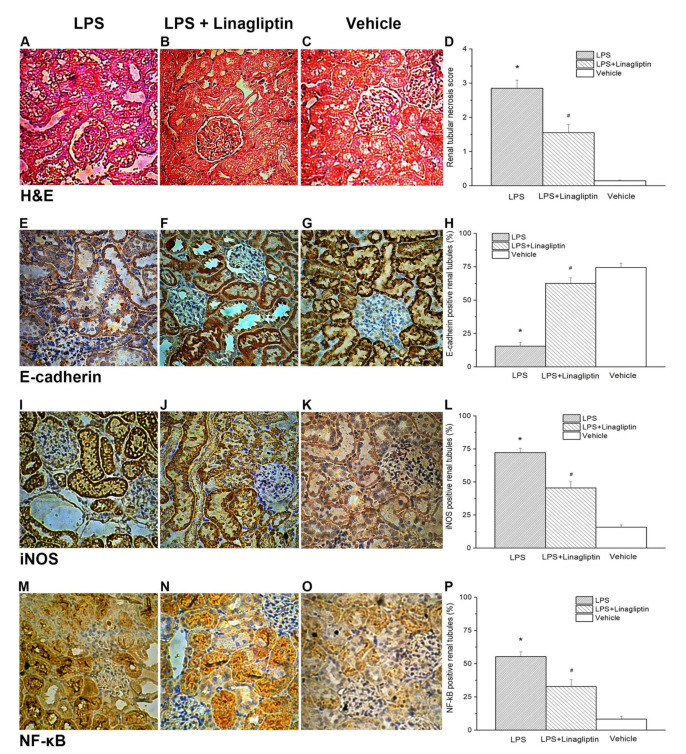
Post-treatment with linagliptin affected the histopathologic changes in the kidneys. Histologic sections stained with hematoxylin and eosin (magnification ×200) from the (**A**) LPS group, (**B**) LPS + Linagliptin group, and (**C**) Vehicle group. Renal tissue injury score after endotoxic-shock-induced acute renal failure in rats (**D**). Immunohistochemical staining for E-cadherin in kidneys (magnification ×200) from the (**E**) LPS group, (**F**) LPS + Linagliptin group, and (**G**) Vehicle group. E-cadherin-positive tubule score after endotoxic-shock-induced acute renal failure in rats (**H**). Immunohistochemical staining for iNOS in kidneys (magnification ×200) from the (**I**) LPS group, (**J**) LPS + Linagliptin group, and (**K**) Vehicle group. Inducible nitric oxide synthase (iNOS)-positive tubule score after endotoxic-shock-induced acute renal failure in rats (**L**). Immunohistochemical staining for NF-κB in kidneys (magnification ×200) from the (**M**) LPS group, (**N**) LPS + Linagliptin group, and (**O**) Vehicle group. NF-κB-positive tubule score after endotoxic-shock-induced acute kidney injury in rats (**P**). * *p* < 0.05 for the LPS group compared with the Vehicle group. # *p* < 0.05 for the LPS + Linagliptin group compared with the LPS group. Vehicle (*n* = 8), LPS (*n* = 4), and LPS + Linagliptin (*n* = 6) groups at 48 h after LPS administration.

To further study how linagliptin improves biochemical profiles in AKI, histopathological examination of the kidney tissue was performed. Endotoxic-shock-induced renal tubular damage, increased expressions of NF-κB and iNOS, and decreased expression of E-cadherin were evident with the H&E stain and IHC stain of the postmortem kidney tissue (Figure 7A,E,I,M). Linagliptin was demonstrated to decrease renal tubular injury scores, suppress NF-κB and iNOS expressions, and preserve E-cadherin expression in the kidney tissues of the endotoxic shock animal model (Figure 7B,F,J,N). The renal tissue injury scores, iNOS-positive cells in the kidneys, and NF-κB-positive cells in the kidneys were significantly lower in the LPS + Linagliptin group after the induction of endotoxic shock compared with those in the LPS group (Figure 7D,L,P). After the induction of endotoxic shock, the LPS + Linagliptin group had significantly greater E-cadherin positivity in the renal tubular cells compared with the LPS group (Figure 7H).

## 3. Discussion

**Figure 8:**
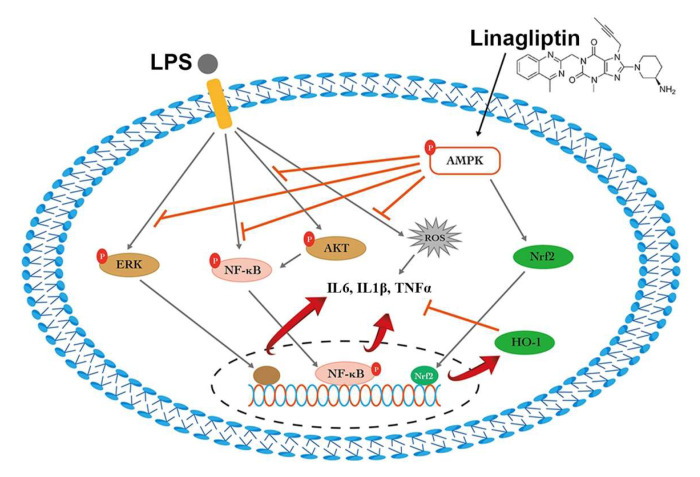
Schematic summary graph of the protective mechanism of linagliptin against lipopolysaccharide (LPS)-induced kidney injury by restoring the AMP-activated protein kinase (AMPK) pathway, nuclear factor erythroid 2-related Figure 2. and heme oxygenase 1 (HO-1) protein, and decreasing pro-inflammatory cytokines (tumor necrosis factor alpha (TNF-α) and interleukin 1 beta (IL-1β, IL-6)), attenuating reactive oxidative species (ROS) overproduction. NFκB, nuclear factor kappa-light-chain-enhancer of activated B cells; AKT, protein kinase B; ERK, extracellular signal-regulated kinases.

In this study, we demonstrated the protective effect of linagliptin against endotoxin-induced kidney injury by restoring the AMPK pathway and HO-1, suppressing pro-inflammatory cytokines such as TNF-⍺ and IL-1β, attenuating ROS overproduction (Figure 8), and reducing renal tubular cell damage, which led to a better survival outcome for the conscious rat model. Although the renal protection of DPP-4 inhibition has been extensively investigated, mostly involving the glucagon-like-peptide-associated glycemic control, the anti-inflammasome effect in the renal fibrosis model [23], mitochondrial homeostasis [24], and anti-proteinuric effects [25] simply represent “pleiotropic” effects [26]. To the best of our knowledge, this is the first study to show evidence of DPP-4 inhibition in the mitigation of S-AKI in animals without diabetes.

Inflammation plays a central role in the development of diabetic kidney disease. Over time, chronic inflammation increases the renal interstitial macrophage and dendritic cell populations, recruits additional inflammatory cells, and further induces the release of numerous pro-inflammatory cytokines (such as IL-1, IL-6, IL-18, and TNF) and chemoattractant molecules (such as CCL2 (also known as MCP1) and CX3C-chemokine ligands 1 (CX3CL1) and 5 (CXCL5)) [27,28]. These inflammatory cells and cytokines are also predominant in sepsis events, albeit in different durations. Through indirect activation of the GLP1-receptor (GLP1R), DPP-4 inhibitor attenuates ROS via HO-1 upregulation and blocks NF-κB p65 from binding to its target genes and the downstream expression of chemokines and cytokines (such as TNF, IL-1β, IL-6, and transforming growth factor-β) [29,30]. These findings could be related to what we found in our study. Further study is warranted to study the statuses of GLP-1 and GLP1R in our model. 

Although endotoxin-induced upregulation of NF-κB has been consistently suppressed by linagliptin in different experiments, including cell models and animal models, evidence of direct interaction between linagliptin and NF-κB or its upstream mechanism is lacking. In addition to the DPP4 status, the cell or plasma level, pharmacologic inhibition, and genetic depletion should be addressed to better understand the underlying molecular mechanism.

## 4. Materials and Methods

### 4.1. Cell Model

NRK-52E cells, which are immortalized rat renal tubular epithelial cell lines, were purchased from the American Type Culture Collection (ATCC; Manassas, VA, USA). Briefly, the cells were passaged every 3–4 days in 100 mm dishes (Falcon, Bedford, MA, USA) using Dulbecco’s Modified Eagle’s Medium-F12 (Sigma-Aldrich, St. Louis, MO, USA) supplemented with 10% fetal bovine serum (Life Technologies Inc., Gaithersburg, MD, USA), insulin–transferrin–sodium selenite media supplement (Sigma-Aldrich, St. Louis, MO, USA), 100 U/mL penicillin, and 100 mg/mL streptomycin (Sigma-Aldrich, St. Louis, MO, USA). For experimental use, these cells were incubated in a humidified atmosphere of 5% CO_2_ and 95% air at 37 °C for 24 h and sub-cultured at 70%–80% confluence. After being washed extensively with phosphate-buffered saline (PBS), the cells were treated with either 0.1 µg/mL linagliptin (Santa Cruz Biotechnology Inc. Dallas, TX, USA. Cat. # sc-364721, Lot # D1921), 10 μg/mL LPS (lipopolysaccharide from Escherichia coli 0111:B4, Sigma-Aldrich, St. Louis, MO, USA, product #L2630, Lot #095M4163V), or LPS + linagliptin for 2 h. The cells were harvested at the end of treatment for molecular analysis. All cellular experiments were repeated three times for semi-quantitative real-time polymerase chain reaction (RT-PCR) and Western blot analysis.

### 4.2. Reactive Oxygen Species (ROS) Evaluation

NRK-52E cells were seeded for 24 h in 12-well microplates with a 5% BS medium to culture 4 × 105 cells. NRK-52E cells were treated separately with 10 μg/mL LPS, 100 μM H_2_O_2_, 0.1 μg/mL linagliptin, and LPS + linagliptin in a serum-free medium for 12 and 24 h. After sample collection, the intracellular ROS level was determined using 2′,7′-dichlorodihydrofluorescein diacetate (DCF-DA) as a fluorescent probe. Then, DNA was marked using 4′,6-diamidino-2-phenylindole dihydrochloride (DAPI). After incubation with 50 μM DCF-DA for 20 min, 0.5 μg/mL DAPI was treated for 1 min. After washing with PBS, DCF-DA and DAPI were evaluated via fluorescence microscopy.

### 4.3. Quantitative Real-Time Polymerase Chain Reaction and Western Blot Analysis

NRK-52E cells were plated at a density of 5 × 10^5^ cells on 6-well culture plates and incubated overnight. Subsequently, the cells were treated with linagliptin (0.1 µg/mL) with or without LPS (10 μg/mL) in a serum-free medium for 12 and 24 h. Total RNA from cells was isolated using TRIzol reagent (GenePure, Chen-Shuo Biotechnology Co.,Ltd New Taipei City, Taiwan) according to the manufacturer’s protocols. The concentration of RNA was determined using a spectrophotometer. Reverse transcription from 2 μg of each pooled RNA sample was performed following the manual of ToolsQuant II Fast RT Kit (KRT-BA06-2, BIOTOOLS). Synthesized cDNA was then diluted to the concentration of 10 ng/μL and stored at −80 °C. TOOLS 2xSYBR qPCR Mix Kit (BIOTOOLS Co., Ltd. New Taipei City, Taiwan, FPT-BB05) was used to perform real-time PCR in SYBR Green I fluorescent-based detection assays, chemically modified HotStar Taq DNA polymerase, and antibody-modified anti-Taq DNA polymerase.

Samples for each target gene were loaded into a 96-well plate in triplicate, along with a triplicate inter-run calibrator and one-single NTC control. CFX Connect Real-Time PCR Detection System (Bio-Rad) was used to perform the two-step real-time PCR, set as follows: 95 °C for 15 min as initial denaturation, then 40 cycles of 95 °C denaturation for 10 s and 62 °C annealing/extension for 30 s, and 95 °C complete extension for 10 s. Melting curve detection was performed from 65 to 95 °C at increments of 0.5 °C every 5 s. The specific primers used for PCR amplification are presented in Supplement Table S1, including NF-κB, chemokine ligand 2 (CCL2), IL-1β, and IL-6. The resulting data were analyzed with CFX MaestroTM Software (Bio-Rad). The relative normalized expression of target genes was calculated according to the 2^−(delta-delta Cq) method [31], using the reference gene β-actin as the internal control, sample “12 h control” for the rest of the groups in 12 h, and sample “24 h control” for the rest of the groups in 24 h.

NRK-52E cells were seeded in a 100 mm culture dish overnight and treated with linagliptin (0.1 µg/mL) with or without 10 μg/mL LPS in a serum-free medium for 12 and 24 h. Moreover, the cells were lysed in a radioimmunoprecipitation assay (RIPA) lysis buffer containing 1 × protease inhibitor (BioBasic Inc., Markham, ON, Canada). After a 12,000× *g* centrifugation for 30 min at 4 °C, the protein content of the supernatant was calculated using a bicinchoninic acid (BCA) protein assay kit (Pierce, Rockford, IL, USA). Protein samples were separated by 8%–12.5% sodium dodecyl sulfate-polyacrylamide gel electrophoresis and transferred to polyvinylidene fluoride membranes (FluoroTrans, PALL, Dreieich, Germany). The membranes were blocked with 5% non-fat dry milk and incubated with primary antibodies overnight at 4 °C. The membranes were washed 3 times with 0.5% Tween-20 in TRIS-buffered saline, incubated with biotin-labeled secondary antibodies (1/1500 dilution; Santa Cruz, CA, USA) for 2 h at room temperature, and then incubated with HRP-conjugated streptavidin for 1 h. Antibody-reactive proteins were interacted by enhanced chemiluminescence (ECL, T-Pro Biotechnology, Taiwan) and detected by using a chemiluminescence/fluorescence imaging analyzer (GE LAS-4000, GE Healthcare Life Sciences, NJ, USA). The band of each sample was quantified using the ImageJ (1.47t) software, and all results were normalized to the value of the control.

### 4.4. Animals

A total of 24 male 14-week-old Wistar–Kyoto rats weighing between 260 and 300 g were obtained from BioLASCO (Taipei, Taiwan) and maintained in a temperature-controlled environment (22–25 °C) in a 12 h/12 h light/dark cycle. Food and water were provided ad libitum.

### 4.5. Blood Vessels Catheterization

On the 8th experimental day, the rats were anesthetized (Matrx VIP 3000, Midmark, Dayton, OH, USA) via 15-min inhalation of isoflurane (Forane, Baxter, Deerfield, IL, USA). The femoral artery and vein were cannulated with polyethylene (PE)-10 catheters. The femoral artery catheter was connected to a pressure transducer to record the arterial pressure and heart rate (HR) using a high-resolution laboratory data recorder (E-corder 410, eDAQ, New Zealand, NSW, Australia). The femoral vein was catheterized for intravenous administration of drugs or fluids. Post-surgery, the rats were awakened and then placed in a metabolic cage. Endotoxic shock was induced 24 h later, while the rats were in the conscious state [32].

### 4.6. Lipopolysaccharide-Induced Endotoxic Shock Rat Model

Endotoxic shock was induced in conscious rats by intravenous injection of Klebsiella pneumoniae lipopolysaccharide (LPS) (Sigma-Aldrich, St. Louis, MO, USA) at a dose of 20 mg/kg (dissolved in 0.5 mL 0.9% normal saline) administered for 30 min. The mean arterial pressure and HR were continuously monitored, and then the animals were sacrificed 48 h after LPS administration.

### 4.7. Experimental Design

As illustrated in Supplemental Figure S1, animals were randomly divided into Vehicle, LPS, and LPS + Linagliptin groups (*n* = 8 in each group). The rats in the LPS group received 20 mg/kg LPS in 0.5 mL normal saline intravenously for 30 min and were then administered 1.0 mL normal saline intravenously for 30 min. The rats in the LPS + Linagliptin group received 20 mg/kg LPS in 0.5 mL normal saline intravenously for 30 min and then were administered 10 mg/kg linagliptin (Fisher Scientific, Pittsburgh, PA, USA) in 1.0 mL normal saline for 30 min [33]. We prepared the linagliptin stock (10 mg/kg linagliptin in 0.9% normal saline) for 8 rats at once. The mixture underwent vigorous vibration till the mixture became a clear-to-slight-hazy suspension. Then, 1.0 mL of the suspension was administered to each rat intravenously for 30 min via a syringe pump, all the time undergoing continuous gentle shaking to keep as homogenous as possible. The rats in the Vehicle group received 0.5 mL normal saline for 30 min and then 1.0 mL normal saline for 30 min intravenously instead of LPS or linagliptin.

### 4.8. Serum Cytokines and Biochemical Assessment

After the animals were sacrificed, blood samples were collected from those that survived the septic shock and centrifuged at 12,000× *g* for 10 min at 4 °C to separate the serum. Serum biochemical measurements of glucose, blood urea nitrogen (BUN), creatinine (Cre), glutamic oxaloacetic transaminase (GOT), glutamic pyruvic transaminase (GPT), lactic dehydrogenase (LDH), and creatine phosphokinase (CPK) were recorded using a biochemistry analyzer (Spotchem SP-4430, Arkray, Minneapolis, MN, USA) [34]. The remaining serum was preserved at −80 °C prior to evaluation of TNF-α and IL-1β using an enzyme-linked immunosorbent assay kit and commercial assay kits (Abcam, Cambridge, MA, USA).

### 4.9. Histopathological and Immunohistochemical Examinations

After the animals were sacrificed, the kidneys were immediately removed and the tissue specimens were fixed overnight in 4% buffered formaldehyde and then processed using standard methods and stained with hematoxylin and eosin (H&E). Tissue analysis was conducted in a blind fashion. Renal tubular injury was scored according to the percentage of tubules in the cortex or the outer medulla demonstrating epithelial necrosis or with the presence of luminal necrotic debris and tubular dilation [35]. Lesions were graded as follows: (0) none; (1) <5%; (2) 5%–25%; (3) 25%–75%; and (4) >75%.

Immunohistochemical examinations were conducted on 3-μm-thick sections. Subsequently, the sections underwent deparaffinization and rehydration. Antigen retrieval was performed with a buffer (Trilogy, Cell Marque, Rocklin, CA, USA) using a microwave oven. After blocking endogenous peroxidase in tissues with a 3% hydrogen peroxide solution for 3 min, the slides were blocked for 1 h with 10% bovine serum albumin (BSA)-containing a buffer at room temperature. The slides were then incubated with anti-E-cadherin mouse monoclonal antibody (1:200 dilution, Abcam, Cambridge, MA, USA), anti-iNOS-1 mouse monoclonal antibody (1:200 dilution, Abcam, Cambridge, MA, USA), and anti-NF-κB mouse monoclonal antibody (1:200 dilution, Abcam, Cambridge, MA, USA) for 15 min at room temperature. Subsequently, the slides were serially rinsed and incubated with biotinylated goat anti-mouse secondary antibodies at room temperature for 30 min. After washing, the slides were incubated in peroxidase-conjugated streptavidin–biotin complex (Dako, Copenhagen, Denmark) for 10 min, followed by a 1–2 min application of 3,3ʹ-diaminobenzidine (DAB). Then, the sections were counterstained with Mayer hematoxylin, dehydrated with ethanol, and coverslipped for evaluation. Positive cells were semi-quantified for immunohistochemistry (IHC) analysis and were evaluated at 10 high-power per section. The data were expressed as a percentage of the positive area examined. All scoring was performed in a blind fashion.

### 4.10. Statistical Analysis

The results are expressed as the mean ± standard error of the mean (SEM). Data were analyzed by one-way analysis of variance with post hoc Bonferroni–Dunn (unless otherwise indicated) for multiple comparisons (SPSS 24.0 for Windows; SPSS, Inc., Chicago, IL, USA). Comparisons between the two groups were made using an unpaired *t*-test. Survival analysis was conducted using the Kaplan–Meier estimator, and comparisons among the mortality rates of the groups were performed using the log-rank test. A *p*-value < 0.05 was considered statistically significant.

## 5. Conclusions

Our study provides insight into the pleiotropic effects of linagliptin in the protection of kidneys against injury from endotoxin-shock-induced catastrophic inflammatory damage by restoring the AMPK pathway and HO-1, suppressing pro-inflammatory cytokines, and attenuating ROS production.

## Supplementary Materials

The following are available online at https://www.mdpi.com/article/10.3390/ijms222011190/s1.
