# Supplementary material for: Linagliptin Protects against Endotoxin-Induced Acute Kidney Injury in Rats by Decreasing Inflammatory Cytokines and Reactive Oxygen Species
Source: Int J Mol Sci. 2021 Oct 17;22(20):11190. doi: 10.3390/ijms222011190 (PMC8540923; doi:10.3390/ijms222011190)
Supplement: Supplementary file 1 [file ijms-22-11190-s001.zip › ijms-1343681-supplementary.pdf]

International Journal of
Molecular Sciences

MDPI

Table S1. Primers used in the quantatitaive real time polymerase chain reaction analyses.

	Forward Primer (5′-3′)	Reverse Primer (5′-3′)
NF-κB	GCTCAAGATCTGCCGAGTAAAC	AGCCAGGTCCCGTGAAATA
CCL2	ACTCACCTGCTGCTACTCATTC	CTACAGCTTCTTTGGGACACCT
IL-1β	GCTAG-TGTGTGATGTTCCCATTAG	CATTGAGGTGGAGAGCTTTCAG
IL-6	CAGAGCAATACTGAAACCCTAG-TTC	TTGGTCCTTAGCCACTCCTT
β-actin	CATTGCTGACAG-GATGCAGAAGG	TGCTGGAAGGTGGACAGTGAGG

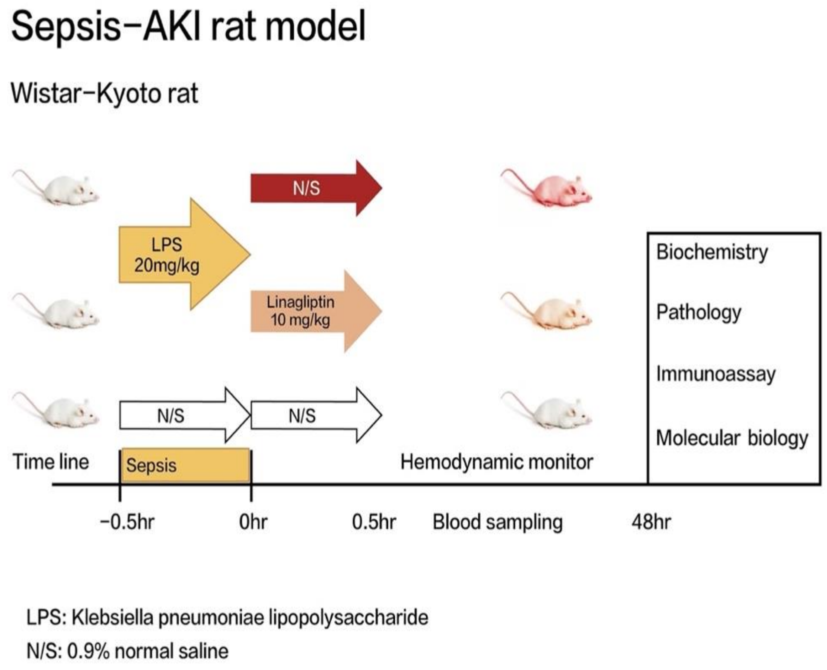

Figure S1 The animal experiment graphic plan**.** LPS, Klebsiella pneumoniae lipopolysaccharide. N/S, 0.9% normal saline.
